# The safety and short‐term outcomes of allogeneic hematopoietic stem cell transplantation with donor vaccination for COVID‐19

**DOI:** 10.1002/mco2.179

**Published:** 2022-10-05

**Authors:** Yihan Ding, Yifan Shen, Yi Fan, Jia Chen, Yang Xu, Depei Wu

**Affiliations:** ^1^ National Clinical Research Center for Hematologic Diseases Jiangsu Institute of Hematology The First Affiliated Hospital of Soochow University Suzhou China; ^2^ Institute of Blood and Marrow Transplantation Collaborative Innovation Center of Hematology Soochow University Suzhou China; ^3^ Department of Hematology The Affiliated Huai'an No. 1 People's Hospital of Nanjing Medical University Huai'an China

Dear Editor,

Coronavirus disease 2019 (COVID‐19), caused by severe acute respiratory syndrome coronavirus‐2 (SARS‐CoV‐2), has spread rapidly across the globe, threatening the health and safety of an increasing number of people. It has been shown that the SARS‐CoV‐2 vaccine is effective in reducing the chance of COVID‐19 infection and the severity of the disease.^1^, ^2^, ^3^ To control the COVID‐19 pandemic, an increasing number of studies are calling for people to receive the SARS‐CoV‐2 vaccine,^4^, ^5^ and the Chinese government is also advocating universal vaccination. Patients with malignant hematologic diseases are mostly immunodeficient,^6^ and recipients are immunosuppressed when undergoing allogeneic hematopoietic stem cell transplantation (allo‐HSCT).^7^ One report on donor‐derived vaccination indicated that vaccination could induce antigen‐specific immune responses in patients after allo‐HSCT.^8^ In addition, most COVID‐19 vaccines in China, different from prevailing types in other countries, are inactivated SARS‐CoV‐2 vaccines. Hitherto, there are no reports on whether the vaccine will induce immune responses impacting immune reconstitution, complications, and outcomes of recipients. Therefore, many clinicians and patients in China hesitate to choose a COVID‐19‐vaccinated person as a donor, which partially inspires us to collect cases of donors who received SARS‐CoV‐2 vaccination to explore the impact of donor vaccination with SARS‐CoV‐2 vaccine on recovery and outcomes after transplantation in recipients.

From March 2021 to December 2021, we retrospectively collected data on a total of 253 patients who received allo‐HSCT at the First Affiliated Hospital of Soochow University, none of them had ever had COVID‐19 prior to transplantation, and none of them were vaccinated for COVID‐19 before transplantation and during the posttransplant follow‐up period. All donors and patients provided written informed consent for the protocol approved by The First Affiliated Hospital of Soochow University's Ethics Committee. We divided the patients into vaccine group (*n* = 82) and control group (*n* = 171) based on whether the donors had received the COVID‐19 vaccine before stem cell collection. In the vaccine group, 88% (72/82) and 12% (10/82) of the donors received inactivated SARS‐CoV‐2 vaccine (Vero Cell) and recombinant SARS‐CoV‐2 vaccine (CHO Cell), respectively (Table S1).

There were no differences between the vaccinated and unvaccinated groups in terms of age, sex, primary disease, pretransplant disease status, or transplantation modality (Table S2). The choice of conditioning regimen did not differ between the two groups (*p* = 0.288), with 90% (74/82) and 89% (152/171) of patients pretreated with busulfan/cyclophosphamide, respectively, while other conditioning regimens included fludarabine/busulfan, total body irradiation/cyclophosphamide, fludarabine/busulfan/melphalan, and fludarabine/amsacrine/cytarabine. Graft‐versus‐host disease (GVHD) prophylaxis in both groups was a strategy that included cyclosporine A/tacrolimus, methotrexate, mycophenolate mofetil, and antithymocyte globulin. Furthermore, there were no differences between the groups in the type of transplant (*p* = 0.510) or the dose of CD34^+^ cells (*p* = 0.075) or mononuclear cells infused (*p* = 0.791). Eighty‐five percent of the vaccine group (70/82) and 88% of the control group (110/171) adopted haploidentical hematopoietic stem cell transplant. Meanwhile, the median CD34^+^ cell infusion counts were 3.74 × 10^6^/kg and 4.21 × 10^6^/kg. For hematopoietic reconstitution, the cumulative incidence of neutrophil engraftment (median 11 days, range 8–17 days vs. median 11 days, range 9–25 days, *p* = 0.882) and platelet engraftment (median 15 days, range 8–61 days vs. median 14 days, range 8–82 days, *p* = 0.713) in the two groups were similar.

For immune reconstitution, we collected data on lymphocytes and analyzed each subpopulation of T cells, B cells, and NK cells at +14d, +1 M, +2 M, +3 M, and +6 M post‐transplantation. We found that the patients in the vaccination group had a lower absolute number of CD3^+^CD4^+^CD25^+^CD127^±^Tregs (0.01 ± 0.30 × 10^6^/L vs. 0.05 ± 0.43 × 10^6^/L; *p* = 0.030) at +14d and a lower absolute number of CD16^+^56^+^ NK cells (0.12 ± 0.09×10^6^/L vs. 0.31 ± 0.30×10^6^/L; *p* = 0.020) at +6 M after transplantation. However, there were similar CD3^+^ T cells, CD3^+^CD4^+^ cells/CD3^+^CD8^+^ cells ratios and CD19^+^ B cells after transplantation (Figure 1A).

**FIGURE 1:**
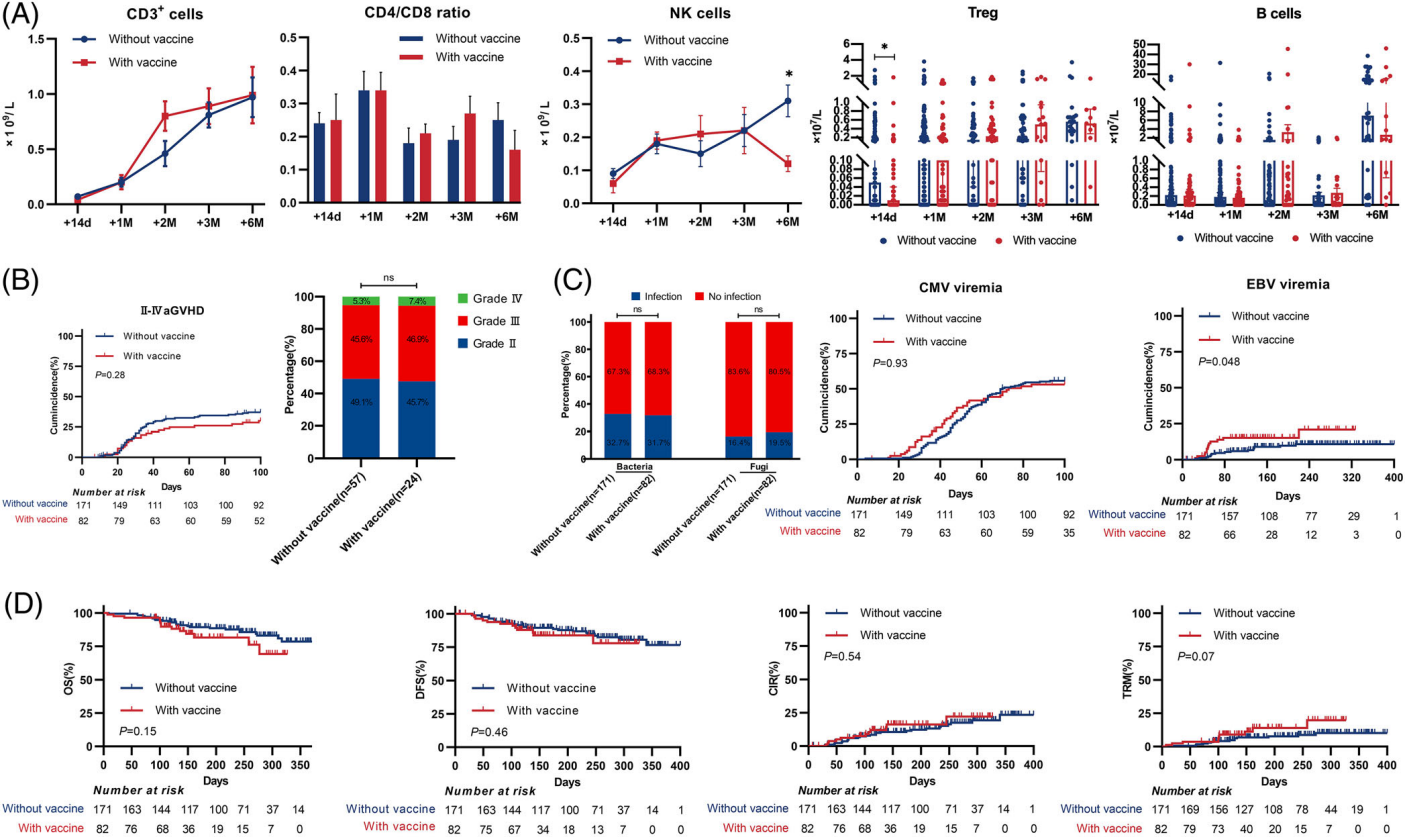
Comparison of the SARS‐CoV‐2 vaccinated group with the unvaccinated group after allogeneic hematopoietic stem cell transplantation (allo‐HSCT). (A) Comparison between subpopulations of lymphocytes in the two groups at 14 days, 1 month, 2 months, 3 months, and 6 months after allo‐HSCT. (B) Comparison of the occurrence of aGVHD in the two groups after allo‐HSCT. (C) Comparison of the incidence of infection in the two groups after allo‐HSCT. (D) Comparison of overall survival (OS), disease‐free survival (DFS), the cumulative incidence of relapse (CIR), and transplantation‐related mortality (TRM) between the two groups at 6 months after allo‐HSCT

We compared the two groups for posttransplantation complications, such as GVHD and infection, as shown in Figure 1B and C. The cumulative incidences of grade II‐IV acute GVHD (aGVHD) were similar between the two groups. The majority of cytomegalovirus (CMV) viremia (95.1%, 137/144) occurred 100 days after transplantation. Therefore, when followed up 100d, the cumulative incidences of CMV viremia were not significantly different between the two groups. Interestingly, the 100‐day incidence of Epstein–Barr virus (EBV) viremia was 14.6% in the vaccine group, which had a higher rate of positivity than that in the control group (14.6% vs. 5.8%, *p* = 0.048). The median time to onset of EBV viremia was 48d (range 24–220) in the vaccine group and 71d (range 24–214) in the control group (*p* = 0.046). The incidences of bacterial and fungal infections that were found to have an etiological basis followed a similar pattern.

With a median follow‐up time of 202 days (range 7–400 days), 12 (14.6%) and 26 (15.2%) relapses were documented in the vaccine and control groups, and 14 (17.1%) and 24 (14.0%) patients died at the last follow‐up, respectively. In the vaccine group, 35.7% of patients died of immune‐related diseases, including four patients died of GVHD and one patient died of other immune‐related organ injuries; the remaining nine patients died of early relapse (4/14), infection (3/14), thrombotic microangiopathy (1/14), and leukoencephalopathy (1/14). In the control group, 45.8% of patients died of relapse (11/24), and the remaining causes of death were infection (8/24), GVHD (4/24), and leukoencephalopathy (1/8). The 6‐month overall survival (85.4% vs. 90.0%, *p* = 0.15) and disease‐free survival (86.6% vs. 89.5%, *p* = 0.46) were similar between the two groups (Figure 1D). The patients whose donors were vaccinated had a similar 6‐month cumulative incidence of relapse compared with that of patients in the control group (13.4% vs. 10.5%, *p* = 0.54). The 6‐month transplantation‐related mortality was 10.9% and 5.3% with and without vaccination, respectively (*p* = 0.07).

There are no reports on whether the COVID‐19 vaccination of donors affects the safety and efficacy of allo‐HSCT. This study preliminarily demonstrated that donors who received the SARS‐CoV‐2 vaccine did not affect the short‐term outcome of allo‐HSCT except for immune reconstitution. We tried to compare the clinical outcome according to the time from vaccination to stem cell collection and whether the full vaccination dosage had been completed, but we did not observe differences because most donors were completely vaccinated. It remains to be explored how the time from inoculation to stem cell collection and whether the full inoculation procedure is completed affects the safety and efficacy of allo‐HSCT.

A study of live‐attenuated varicella zoster vaccine noted that donor vaccination is safe, but whether it confers significant protection for patients requires further study.^9^ Bogeholz et al.^10^ indicated in a study of measles, mumps, and rubella antibody titers in allo‐HSCT recipients that donor immune status did not appear to have an influence on antibody protection after HSCT. During our observation period, no patients were found with SARS‐CoV‐2 infection after transplantation, this probably be related to the absence of the COVID‐19 epidemic in the area of our center. Notably, in this study, we only analyzed the short‐term outcomes with allo‐HSCT patients with donor vaccination with the SARS‐CoV‐2 vaccine, and the long‐term outcomes need to be further studied.

In conclusion, pre‐HSCT vaccination of the donor with the SARS‐CoV‐2 vaccine does not affect the short‐term safety and efficacy of allo‐HSCT, and well‐designed prospective studies are needed to determine whether it influences the long‐term clinical outcome and provides protection to the patient.

## AUTHOR CONTRIBUTIONS

YX and DW designed the study. YD, YS, and YF contributed to the collection of data. YS analyzed the data. YD and JC discussed and interpreted the results. YD wrote the manuscript. All authors contributed to the article and approved the submitted version.

## CONFLICTS OF INTEREST

The authors declare no conflicts of interest to report.

## ETHICS STATEMENT

This study was approved by the ethics committee of the First Affiliated Hospital of Soochow University and was conducted in accordance with the Declaration of Helsinki. Informed consent was obtained from all patients and donors before data collection.

## Supporting information

Supporting information

## Data Availability

The datasets generated during and/or analyzed during the current study are available from the corresponding author on reasonable request.
