# Supplementary material for: The safety and short‐term outcomes of allogeneic hematopoietic stem cell transplantation with donor vaccination for COVID‐19
Source: MedComm (2020). 2022 Oct 5;3(4):e179. doi: 10.1002/mco2.179 (PMC9534375; doi:10.1002/mco2.179)
Supplement: Supplementary file 1 — Supporting information [file MCO2-3-e179-s001.docx]

Supplementary Materials

**The safety and short-term outcomes of allogeneic hematopoietic stem cell transplantation with donor vaccination for COVID-19**

Yihan Ding ^1,2,3#^, Yifan Shen^1,2#^, Yi Fan^1,2#^, Jia Chen^1,2^, Yang Xu^1,2*^, Depei Wu^1,2*^

^1^National Clinical Research Center for Hematologic Diseases, Jiangsu Institute of Hematology, The First Affiliated Hospital of Soochow University, Suzhou, China.

^2^Institute of Blood and Marrow Transplantation, Collaborative Innovation Center of Hematology, Soochow University, Suzhou, China.

^3^Department of Hematology, The Affiliated Huai'an No.1 People's Hospital of Nanjing Medical University, Huai'an, China.

**Study subjects**

Data were collected from 253 patients who received allogeneic hematopoietic stem cell transplantation (allo-HSCT) at the First Affiliated Hospital of Soochow University from March 2021 to December 2021. We reviewed the data of these patients for retrospective analysis. This study was approved by the ethics committee of the First Affiliated Hospital of Soochow University and was conducted in accordance with the Declaration of Helsinki.

**Inclusion and exclusion criteria**

The criteria for inclusion were as follows: 1) The patient was treated with allo-HSCT. 2) The patient has not received any vaccination, including the COVID-19 vaccine, within six months prior to allo-HSCT. 3) The patients were not infected with COVID-19 prior to transplantation. The exclusion criteria were as follows: 1) The donor has received vaccines other than COVID-19 within six months prior to stem cell collection. 2) The donor had COVID-19 before the stem cell collection. 3) The donor or patient has a clear history of viral or fungal infection within one month prior to transplantation.

**Immunosuppressive regimens**

Immunosuppressive regimens were the same between the vaccinated and unvaccinated groups, including cyclosporin A (CsA)/Tacrolimus (FK506), methotrexate (MTX), mycophenolate mofetil (MMF) and ATG. CsA at 3 mg/kg/d was given by continuous infusion over 24 hours since day -10 and ended when patients with a target blood concentration ranging from (200-300) ng/mL switched to oral intake. FK506 at 0.03 mg/kg/d was used as a precautionary measure against CsA intolerance. MTX was given at 15 mg/kg/d on day +1 and 10 mg/kg/d on days +3, +6, and +11. From day -10 to day +30, patients took 500 mg MMF orally twice daily. ATG was administered intravenously at 2.5 mg/kg on days -5 to -2.

**SARS-CoV-2 vaccine**

The SARS-CoV-2 vaccine (Vero Cell) is manufactured by Sinovac Research & Development Co., Ltd. It is a preparation made from a novel coronavirus (strain CZ02) grown in the kidney cell cultures (Vero Cell) of the African green monkey^1^. A dose of 0.5 mL contains 600 SU of SARS-CoV-2 virus antigen. The basal immunization was 2 doses at intervals ≥3 weeks and <8 weeks. A booster immunization can be given 6 months after the completion of the basic immunization.

The SARS-CoV-2 vaccine (CHO Cell) is manufactured by Zhifei Longcom Biopharmaceutical Co., Ltd. It is a preparation made from recombinant CHO cells expressing novel coronavirus spike-in glycoprotein receptor binding region NCP-RBD protein (https://www.zflongkema.com/d/file/product/ybcp/2022-03-24/6a35d50f8ddf66a326490f92fad510cb.pdf). A dose of 0.5 mL contains 25 μg of NCP-RBD protein. Basic immunization is 3 doses at 1-month intervals.

**Statistical Analysis**

Nonparametric variables were analyzed by the chi-square test or Mann–Whitney test. Comparisons of categorical variables were determined by Pearson's chi-squared test or Fisher's exact test. OS and DFS were estimated by the Kaplan–Meier method and log-rank test. TRM and CIR were estimated by Gray's test and each event was considered a competing risk. A P value < 0.05 was considered statistically significant. SPSS 26.0 and R 4.0.2. were used for statistical analyses.

**References:**

1. Wu Z, Hu Y, Xu M, et al. Safety, tolerability, and immunogenicity of an inactivated SARS-CoV-2 vaccine (CoronaVac) in healthy adults aged 60 years and older: a randomised, double-blind, placebo-controlled, phase 1/2 clinical trial. *Lancet Infect Dis*. 2021;21(6):803-812.

**Supplementary Tables**

TABLE S1 | Vaccination status of donors

| **Type of SARS-CoV-2 vaccine and times of inoculation before stem cell collection, n (%)** | **N (total: 82)** | **Median time from last vaccination to allo-HSCT, days (range)** |
| --- | --- | --- |
| **Vero cell**, n (%) | 72 (87.8) |  |
| 1 | 13 (18.1) | 70 (28-170) |
| 2 | 52 (72.2) | 90 (7-253) |
| 3 | 1 (1.4) | 32 |
| Unknown | 6 (8.3) |  |
| **CHO cell**, n (%) | 10 (12.2) |  |
| 1 | 1 (10.0) | 46 |
| 2 | 4 (40.0) | 38.5 (24-77) |
| 3 | 3 (30.0) | 111 (83-123) |
| Unknown | 2 (10.0) |  |

TABLE S2 | Clinical characteristics of patients and donors

| **Variable** | **With vaccination** | **Without vaccination** | ***P*** |
| --- | --- | --- | --- |
| **Number** (total: 251) | 82 | 171 |  |
| **Patient gender**, n (%) |  |  | 0.149 |
| Male | 44 (53.7) | 108 (63.2) |  |
| Female | 38 (46.3) | 63 (36.8) |  |
| **Patient age** (years), median (range) | 38 (15-65) | 39 (13-75) | 0.715 |
| **Donor gender**, n (%) |  |  |  |
| Male | 56 (68.3) | 127 (74.3) | 0.320 |
| Female | 26 (31.7) | 44 (25.7) |  |
| **Donor age** (years), median (range) | 33 (12-62) | 32 (7-66) | 0.109 |
| **Type of disease and disease status at allo-HSCT**, n (%) |  |  | 0.258 |
| AML, n (%) | 39 (47.6) | 76 (44.4) | 0.817 |
| CR | 32 (82.1) | 61 (80.3) |  |
| No CR | 7 (17.9) | 15 (19.7) |  |
| MDS and CMML, n (%) | 10 (12.1) | 27 (15.8) | 0.992 |
| CR | 7 (70.0) | 17 (63.0) |  |
| No CR | 3 (30.0) | 10 (37.0) |  |
| ALL, n (%) | 23 (28.0) | 34 (19.9) | >0.999 |
| CR | 20 (87.0) | 29 (85.3) |  |
| No CR | 3 (13.0) | 5 (14.7) |  |
| HAL, n (%) | 0 (0) | 4 (2.3) |  |
| CR | 0 (0) | 4 (100.0) |  |
| No CR | 0 (0) | 0 (0) |  |
| Lymphoma, n (%) | 7 (8.5) | 11 (6.4) | >0.999 |
| CR | 5 (71.4) | 8 (72.7) |  |
| No CR | 2 (28.6) | 3 (27.3) |  |
| MPN | 0 (0) | 4 (2.3) |  |
| AA | 3 (3.7) | 15 (8.8) |  |
| **Conditioning regimen**, n (%) |  |  | 0.288 |
| Bu/Cy | 74 (90.2) | 152 (88.9) |  |
| Flu/Bu | 2 (2.4) | 1 (0.6) |  |
| TBI/Cy | 5 (6.1) | 8 (4.7) |  |
| FBM | 1 (1.2) | 9 (5.3) |  |
| FLAMSA | 0 (0) | 1 (0.6) |  |
| **Type of transplant**, n (%) |  |  | 0.510 |
| Haplo-HSCT | 70 (85.4) | 151 (88.3) |  |
| MSD-HSCT | 12 (14.6) | 20 (11.7) |  |
| **Stem cell source**, n (%) |  |  | 0.838 |
| PB | 49 (59.8) | 102 (59.6) |  |
| BM | 3 (3.7) | 4 (2.3) |  |
| PB+BM | 30 (36.6) | 65 (38.0) |  |
| **MCN** (×10^8^/kg), median (range) | 10.33 (3.58-24.36) | 9.85 (3.65-39.24) | 0.791 |
| **CD34** (×10^6^/kg), median (range) | 3.74 (1.6-9.78) | 4.21 (0.59-12.19) | 0.075 |
| **Neutrophil Engraftment**, n (%) |  |  | 0.246 |
| Engrafted | 80 (97.6) | 170 (99.4) |  |
| NE>0.5×10^9^/L(d), median(range) | 11 (8-17) | 11 (9-25) | 0.882 |
| Graft failure | 2 (2.4) | 1 (0.6) |  |
| **Platelet Engraftment**, n (%) |  |  | 0.271 |
| Engrafted | 61 (74.4) | 143 (83.6) |  |
| PLT>20×10^9^/L(d), median(range) | 15 (8-61) | 14(8-82) | 0.713 |
| Graft failure | 8 (9.8) | 11 (6.4) |  |
| Unknown | 13 (15.9) | 17 (9.9) |  |

Abbreviation: allo-HSCT, allogeneic hematopoietic stem cell transplantation; AML, acute myeloblastic leukemia; MDS, myelodysplastic syndrome; CMML, chronic myelomonocytic leukemia; ALL, acute lymphoblastic leukemia; HAL, hybrid acute leukemia; MPN, myeloproliferative neoplasms; AA, aplastic anemia; CR, complete remission; Bu/Cy, busulfan/cyclophamide; Flu/Bu, fludarabine/ busulfan; TBI/Cy, total body irradiation/cyclophamide; FBM, fludarabine/busulfan/melphalan; FLAMSA, fludarabine/amsacrine/cytarabine; Haplo-HSCT, haploidentical hematopoietic stem cell transplantation; MSD-HSCT, matched sibling donor hematopoietic stem cell transplantation; PB, peripheral blood; BM, bone marrow; MCN, mononuclear cells; NE, neutrophil; PLT, platelet.
